# Global Characterization of Differential Gene Expression Profiles in Mouse Vγ1^+^ and Vγ4^+^ γδ T Cells

**DOI:** 10.1371/journal.pone.0112964

**Published:** 2014-11-18

**Authors:** Peng Dong, Siya Zhang, Menghua Cai, Ning Kang, Yu Hu, Lianxian Cui, Jianmin Zhang, Wei He

**Affiliations:** 1 Department of Immunology, Institute of Basic Medical Sciences, Chinese Academy of Medical Sciences and School of Basic Medicine, Peking Union Medical College, State Key Laboratory of Medical Molecular Biology, Beijing, China; 2 Neuroregeneration and Stem Cell Programs, Institute for Cell Engineering, Department of Neurology, Johns Hopkins University School of Medicine, Baltimore, MD, United States of America; Instituto de Medicina Molecular, Portugal

## Abstract

Peripheral γδ T cells in mice are classified into two major subpopulations, Vγ1^+^ and Vγ4^+^, based on the composition of T cell receptors. However, their intrinsic differences remain unclear. In this study, we analyzed gene expression profiles of the two subsets using Illumina HiSeq 2000 Sequencer. We identified 1995 transcripts related to the activation of Vγ1^+^ γδ T cells, and 2158 transcripts related to the activation of Vγ4^+^ γδ T cells. We identified 24 transcripts differentially expressed between the two subsets in resting condition, and 20 after PMA/Ionomycin treatment. We found that both cell types maintained phenotypes producing IFN-γ, TNF-α, TGF-β and IL-10. However, Vγ1^+^ γδ T cells produced more Th2 type cytokines, such as IL-4 and IL-5, while Vγ4^+^ γδ T cells preferentially produced IL-17. Our study provides a comprehensive gene expression profile of mouse peripheral Vγ1^+^ and Vγ4^+^ γδ T cells that describes the inherent differences between them.

## Introduction

γδ T cells were discovered more than 30 years ago. Although considerable progress has been made in characterizing their biological significance, much remains unknown. γδ T cells arise earlier than αβ T cells during thymic ontogeny, predominately at the early stage of fetal development [1]. After birth, however, γδ T cells make up a minor fraction of circulating T lymphocytes in rodents and humans. Similar to αβ T cells, γδ T cells also have a diverse repertoire of T cell receptors (TCR) derived through somatic rearrangement of V, D and J gene segments. Although few V, D and J gene elements are responsible for genetic rearrangement, additional diversity is added to the γ and δ chains via junctional diversification processes [2].

γδ T cells exert diverse functions, however, individual subsets within the population appear to be biased toward specialized functions [1]. Mouse peripheral lymphoid γδ T cells are classified into two major subsets, Vγ1^+^ and Vγ4^+^ γδ T cells, depending on their TCR expression [1], [3], [4]. Vγ1^+^ and Vγ4^+^ γδ T cells perform distinct functions in many disease models. For example, Vγ1^+^ γδ T cells produce IL-4 and IFN-γ in the liver [5], and Vγ4^+^ γδ T cells produce IFN-γ or IL-17 depending on the studied models [6]. Vγ1^+^ and Vγ4^+^ γδ T cells function as oppositional pairs in diseases including coxsackievirus B3 infection [7], West Nile virus infection [4], airway hyperresponsiveness [8], [9], macrophage homeostasis [10] and ovalbumin induced IgE production [11]. However, the functional relatedness of Vγ1^+^ and Vγ4^+^ γδ T cells remains unresolved, partly due to a lack of comprehensive analysis and comparison of gene expression. Although, gene-expression profiles of emergent γδTCR^+^ thymocytes have been reported [12], a comprehensive analysis of peripheral Vγ1^+^ and Vγ4^+^ γδ T cells functional differences has not been reported. This is likely due to the limited number of cells that can be obtained from healthy mice.

In this study, we expanded Vγ1^+^ and Vγ4^+^ γδ T cells simultaneously from the same pool of mouse splenocytes. We comprehensively analyzed gene expression profiles using Illumina’s sequencing technology. We identified 1995 transcripts related to the activation of Vγ1^+^ γδ T cells, and 2158 transcripts were related to the activation of Vγ4^+^ γδ T cells. Interestingly, only 24 transcripts were differentially expressed between two subsets in resting condition, and 20 transcripts after PMA/Ionomycin-induced activation. Both cells produced high levels of IFN-γ, TNF-α, TGF-β and IL-10. However, Vγ1^+^ γδ T cells produced more Th2 type cytokines, while Vγ4^+^ γδ T cells tended to produce more IL-17. These findings describe the inherent differences between Vγ1^+^ and Vγ4^+^ γδ T cells.

## Materials and Methods

### Mice

Male C57BL/6J mice aged 6–8 weeks were purchased from the National Institute for Food and Drug Control. All mice were maintained under specific pathogen-free conditions in the Experimental Animal Center, Institute of Basic Medical Sciences, Chinese Academy of Medical Sciences. All animal experiments were approved by and performed in accordance with the guidelines of the international Agency for Research on Cancer’s Animal Care and Use Committee and IBMS/PUMC’s Animal Care and Use Committee.

### Expansion of Vγ1^+^ and Vγ4^+^ γδ T cells

Vγ1^+^ and Vγ4^+^ γδ T cells were expanded from splenocytes as described previously [13]. Briefly, flat-bottom 24 well plates were coated with 500µl purified anti-mouse TCRγ/δ antibody (UC7–13D5, 1µg/ml; Biolegend) at 37°C for 2 hours. Splenocytes were collected from six male C57BL/6J mice to decrease individual variation. Erythrocytes were lysed in Tris-NH4Cl buffer. Cells were then loaded onto a sterile nylon wool column, sealed and incubated at 37°C with 5% CO2 for 45 minutes. 5×10^7^ cells were eluted and added to the Ab-coated wells (4×10^6^ cells/well) and cultured in RPMI 1640 medium (Gibco BRL) supplemented with 10% fetal calf serum and IL-2 (200 IU/ml). After 8 days of expansion, the proportion of γδ T cells reached approximately 80% as determined by Flow Cytometry.

### Cell sorting and stimulation

1.0×10^7^ Vγ1^+^ and 1.2×10^7^ Vγ4^+^ γδ T cells were sorted by Flow Cytometric Cell Sorting (FACS) with PE conjugated anti-mouse TCR Vγ1.1/Cr4 antibody (2.11, Biolegend) and APC conjugated anti-mouse TCR Vγ2 antibody (UC3–10A6, Biolegend). The purity of sorted cells was more than 99%. 5×10^6^ cells per well were seeded into 6-well culture plates at a concentration of 1×10^6^/ml and rested overnight at 37°C in 5% CO2 in RPMI with 10% FCS. Cells were stimulated for 4 h with PBS or 20 ng/ml of PMA (Sigma) and 0.5µg/ml of Ionomycin (Sigma). Cells were washed with PBS and pelleted by centrifugation. Total RNA from each sample was extracted by Trizol reagent (Invitrogen) according to the manufacturer’s instructions. The quality of total RNA from each sample was confirmed and comparable, based on results of Agilent Technologies 2100 Bioanalyzer.

### Processing samples for Illumina sequencing

We prepared the Illumina libraries according to the manufacturer’s instructions. Briefly, mRNAs were extracted from total RNA by mRNA enrichment kit (Life technologies, USA) followed by fragmentation of mRNA into 250–350 bp sizes. The first strand cDNAs were synthesized using reverse transcriptase and random primers. Second strand cDNAs were synthesized using DNA Polymerase I followed by the addition of a single A base at the ends for the ligation to the adapters. After purification, the final cDNA library was created by PCR. Finally, 400–500 bp products were used for cluster generation, 36 bp single-end sequencing was performed using Illumina HiSeq 2000 Sequencer according to the manufacturer’s instructions (Beijing Berry Genomics Co. Ltd. China). The RNA-Seq raw data files have been deposited in NCBI’s Sequence Read Archive (SRA) and are accessible through SRA Series accession number SRP042029.

### Analysis of RNA-seq data

We performed base calling using CASAVA 1.7 software (Illumina). Low quality and polluted adapter reads were filtered; clean reads were stored on fastq files. The sequence reads were aligned to the mouse genome (mm9), and gene expression was calculated by RPKM value. Differentially expressed transcripts were identified using General Chi-square test analysis. Q values were obtained by the “BH” method [14]. NIH DAVID web server was used for the functional annotation clustering analysis of differentially expressed transcripts.

### Quantitative RT-PCR

Several genes from Vγ1^+^ and Vγ4^+^ γδ T cells were selected for verification from biological replicates with real-time quantitative PCR. RNA was extracted as described above. 500 ng of total RNA was reverse transcribed using PrimeScript RT reagent Kit with gDNA Eraser (Takara Bio). Gene-specific primers are listed in (Table 1). The real-time quantitative PCR was performed on the StepOnePlus Real-Time PCR System (Life Technologies) using SYBR green labeling (SYBR Premix Ex Taq II; Takara Bio). A cycle threshold (Ct) was assigned at the beginning of the logarithmic phase of PCR amplification and relative quantitation was done using the 2^−ΔΔCt^ method. β-actin was used for normalization control.

**Table 1:** Gene-specific primers for real-time quantitative PCR.

Specificity	Primer orientation	Sequence (5′ → 3′)
IL-4	Forward	ACGGAGATGGATGTGCCAAAC
	Reverse	AGCACCTTGGAAGCCCTACAGA
IL-5	Forward	TGAGGCTTCCTGTCCCTACTCATAA
	Reverse	TTGGAATAGCATTTCCACAGTACCC
IL-17A	Forward	CTGATCAGGACGCGCAAAC
	Reverse	TCGCTGCTGCCTTCACTGTA
IL-17F	Forward	ATGAAGTGCACCCGTGAAACAG
	Reverse	CTCAGAATGGCAAGTCCCAACA
SCART 2	Forward	GGATCAGGGCCTTTGTGGA
	Reverse	TGCCATTGACCAGTCGGAAC
beta-actin	Forward	CATCCGTAAAGACCTCTATGCCAAC
	Reverse	ATGGAGCCACCGATCCACA

### Cytokines

Cells were stimulated 4 h with PBS or PMA and Ionomycin then pelleted by centrifugation. Determined cytokine concentration in cell-free supernatants by enzyme linked immunosorbent assay (ELISA; R&D Systems) as described previously [15] and MILLIPLEX MAP Mouse Cytokine Kit (MT17MAG47 K–PX25; Merck Millipore) according to the manufacturer’s instructions.

## Results

### Expansion and isolation of Vγ1^+^ and Vγ4^+^ γδ T cells from mouse splenocytes

**Figure 1:**
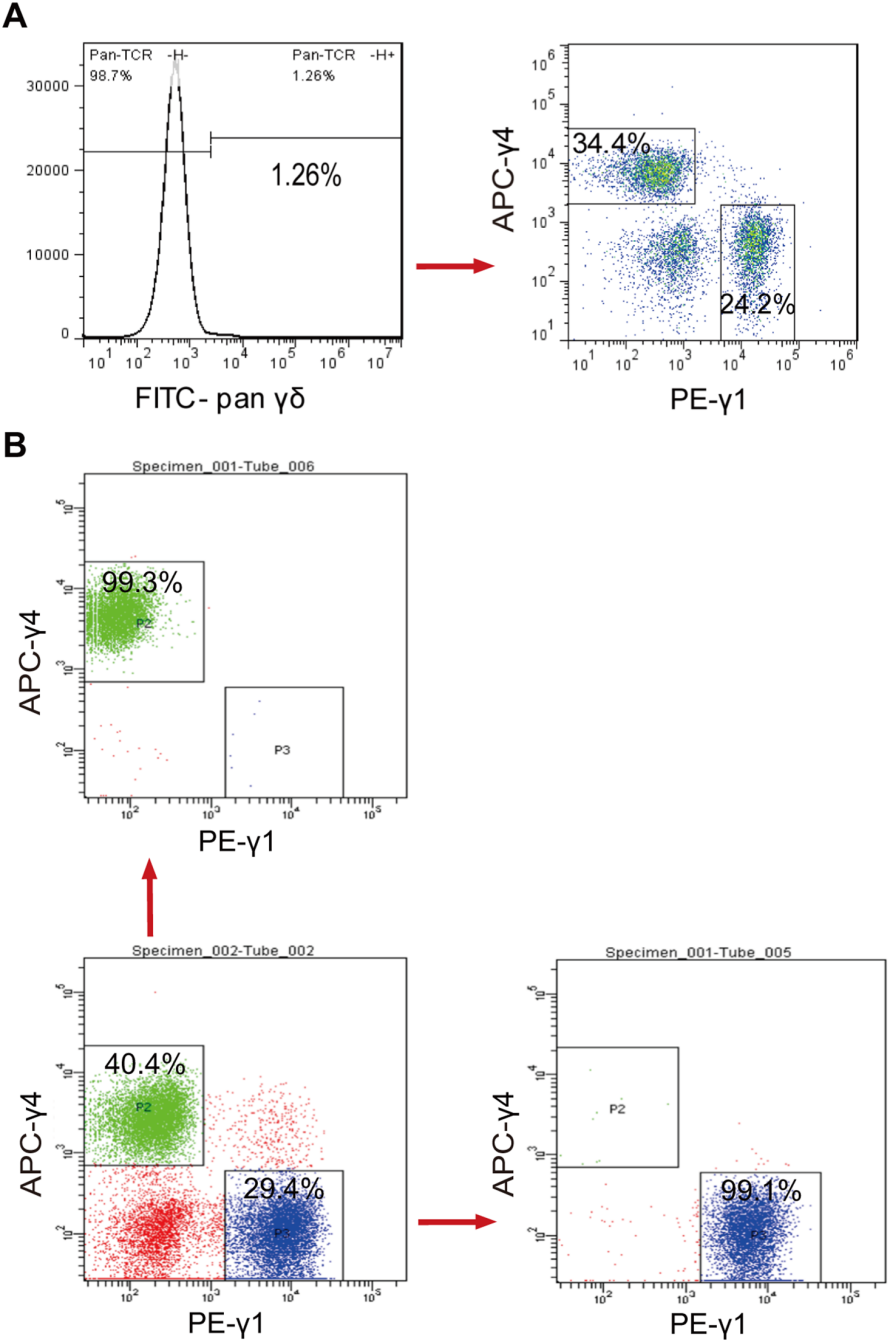
Vγ1+ and Vγ4+ γδ T cells are the major subpopulations in the spleen. (A) γδ T cells account for approximately 1.5% of total splenocytes. Isolated fresh Vγ1^+^ and Vγ4^+^ γδ T cells comprised approximately 35% and 25% of γδ T cells respectively. (B) 1.5×10^8^ cells were expanded simultaneously from a single pool of mouse splenocytes with purified pan anti-mouse TCR γδ antibody (UC7–13D5). After 8 days, Vγ1^+^ and Vγ4^+^ γδ T cells comprised approximately 40% and 30% of the expanded cells, respectively. 1.0×10^7^ Vγ1^+^ γδ T cells were sorted by FACS with PE conjugated anti-mouse TCR Vγ1.1/Cr4 antibody and 1.2×10^7^ Vγ4^+^ γδ T cells were sorted by FACS with APC conjugated anti-mouse TCR Vγ2 antibody. Purity of sorted cells was >99%. Data are representative of four independent experiments.

γδ T cells account for approximately 1∼2% of total splenocytes in healthy mice and Vγ1^+^ and Vγ4^+^ γδ T cells comprised approximately 35% and 25% respectively (Figure 1A). Therefore, we expanded the cells from mouse spleens *in vitro* for RNA-seq analysis. Although Vγ1^+^ and Vγ4^+^ γδ T cells can be expanded separately with sorted splenic γδ T cells using anti-Vγ1 and anti-Vγ4 Abs [16], [17], potentially important biological interactions between the subsets during culture would be neglected. We therefore established a primary culture method to expand the cells simultaneously from the same pool of mouse splenocytes with pan anti-mouse TCR γδ antibodies (UC7–13D5) and IL-2. After 8 days of expansion, the proportion of γδ T cells reached approximately 80%, Vγ1^+^ and Vγ4^+^ γδ T cells comprised approximately 40% and 30% of the expanded cells, respectively (Figure 1B). No significant change was observed in the ratio of γ1 cells to γ4 cells in the *in vitro* expanded γδ T cells when compared with that of freshly isolated γδ T cells (*in vivo* subsets). γδ T cells were not screwed to one preferential subset after in vitro expansion, suggesting that *in vitro* expanded γδ T cells with anti-mouse TCRγδ antibodies plus IL-2 were still representative of *in vivo* subsets of γδ T cells. Expanded Vγ1^+^ and Vγ4^+^ γδ T cells were then sorted by FACS with PE-conjugated anti-mouse TCR Vγ1.1/Cr4 antibody and APC conjugated anti-mouse TCR Vγ2 antibody. We found the purities of sorted Vγ1^+^ and Vγ4^+^ γδ T cells were more than 99% (Figure 1B).

### cDNA library preparation for RNA sequencing from resting and activated Vγ1^+^ and Vγ4^+^ γδ T cells

**Figure 2:**
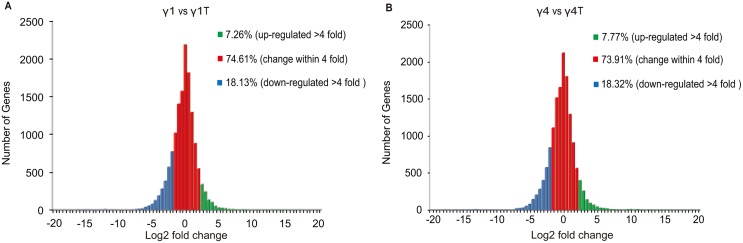
The distribution of gene expression. The ‘x’ axis represents Log fold-change of differentially expressed genes. The ‘y’ axis represents number of genes. Red region represents genes with expression within 4-fold change; green and blue regions represent genes with more than 4-fold change either up or down regulated, respectively. Library pairs: A, resting Vγ1^+^ vs activated Vγ1^+^ γδ T cells; B, resting Vγ4^+^ vs activated Vγ4^+^ γδ T cells.

In order to compare gene expression profiles between subsets in both the resting and activated state, sorted cells were rested overnight at 37°C then stimulated 4 h with either PBS (control) or 20 ng/ml of PMA+0.5 µg/ml of Ionomycin (activated) before mRNA extraction and fragmentation. After cDNA synthesis, adapter ligation and PCR amplification, four cDNA libraries were constructed for the resting and activated γδ T cell subsets. 400–500 bp-sized products were used for cluster generation and 36 bp single-end sequencing was performed by using Illumina HiSeq 2000 Sequencer. Approximately 28 million clean reads were obtained from each sample. More than 88% of reads were mapped to the mouse genome using the default setting in TopHat, suggesting high quality of RNA-seq (Table 2). Cufflinks with default settings were used to assemble the mapped reads against the ENSEMBL gene structure annotation, and estimated expression levels for each transcript. More than 18,286 genes were detected. 25.4–26.1% of genes showed expression levels changed by at least four fold while the majority of genes changed less than four fold (Figure 2, Dataset S1).

**Table 2:** Sequencing reads and mapping rates of each sample.

Sample Info	Total Reads	Mapped Reads	Ratio
γ1-PBS	22,672,055	20,190,322	89.05%
γ1-PMA/Ion	28,235,208	24,381,541	86.35%
γ4-PBS	33,529,245	29,854,538	89.04%
γ4-PMA/Ion	34,338,657	30,274,218	88.16%

γ1-PBS, Vγ1^+^ γδ T cells treated with PBS; γ1-PMA/Ion, Vγ1^+^ γδ T cells treated with PMA and Ionomycin; γ4-PBS, Vγ4^+^ γδ T cells treated with PBS; γ4-PMA/Ion, Vγ4^+^ γδ T cells treated with PMA and Ionomycin.

### Differential gene expression between Vγ1^+^ and Vγ4^+^ γδ T cells

RNA-seq results show Vγ1^+^ and Vγ4^+^ γδ T cells share similar transcript profiles in both the resting and activated subsets. We identified 24 transcripts with differential expression between the resting Vγ1^+^ and Vγ4^+^ γδ T cells (Table 3). We used the Database for Annotation, Visualization and Integrated Discovery (DAVID), an on-line functional annotation tool for gene enrichment analysis, to gain further insight into biological pathways associated with the differentially expressed gene transcripts. We found most of the differentially expressed genes in the resting subsets related to chemokines, transcription and the plasma membrane (Table 3). Resting Vγ1^+^ γδ T cells expressed higher levels of XCL1 and CCL1 compared with Vγ4^+^ γδ T cells, suggesting Vγ1^+^ γδ T cells possess higher chemotactic activity for lymphocytes and monocytes. Vγ4^+^ γδ T cells displayed higher levels of *Rorc*, *Sox13* and *Scart2* expression. In addition, high levels of *Bclaf1* and *Atf2* were expressed in Vγ4^+^ γδ T cells while *Arnt2*, *Hmga1* and *Zfp386* were preferentially expressed in Vγ1^+^ γδ T cells.

**Table 3:** 24 transcripts expressed differently between resting Vγ1^+^ and Vγ4^+^ γδ T cells.

Category	GN	AN	γ1 RPKM	γ4 RPKM	Gene function
Chemokine					
	XCL1	NM_008510	340.582	59.158	Chemotactic activity
	CCL1	NM_011329	84.8039	11.7718	Chemotactic activity
Transcription					
	*BCLAF1	NM_001025392	0.10265	8.85303	Transcriptional repressor
	RORC	NM_011281	0.0844492	0.761408	Orphan nuclear receptor
	SOX13	NM_011439	0.909818	4.99875	Ttranscription factor
	*ATF2	NM_009715	0.702722	4.69872	Transcriptional activator
	*ARNT2	NM_007488	0.799234	0.135113	Recognizes xenobiotic response element (XRE)
	*HMGA1	NM_001039356	5.3784	0.84425	Regulation of inducible gene transcription
	*ZFP386	NM_019565	20.4163	0.927052	Transcriptional regulation
Plasma membrane					
	*CD74	NM_001042605	3.99813	0.540024	Antigen processing
	*CTC1	NM_001013256	0.062176	6.89298	Uncharacterized
	*ABI1	NM_145994	0.0359843	6.83637	Cytoskeletal reorganization and EGFR signaling
	*CACNB3	NM_001044741	0.410331	4.17596	The beta subunit of calcium channels
	*SYT13	NM_183369	1.88579	0.141228	Vesicle trafficking
	*SLC17A6	NM_080853	0.80135	0.0264333	Mediates the uptake of glutamate
	*TMEM219	NM_028389	0.071877	43.5973	Unknown
Miscellaneous					
	SCART2	NM_175533	0.22478	3.02098	Scavenger receptor
	*SENP7	NM_001003972	0.178749	7.79658	Protease
	*ENTPD5	NM_007647	8.35339	0.00516434	Promote reglycosylation
	*FAR1	NM_026143	0.900286	6.64512	Fatty Acyl CoA Reductase 1
	*GOLGA2	NM_133852	0.942965	7.61176	Maintaining cis-Golgi structure
	*ITIH5	NM_172471	2.98889	0.530132	Tumor suppressor
	*PPHLN1	NM_001083114	4.76635	0.114948	Epidermal integrity and barrier formation
	*BC003331	NM_001077237	5.03977	0.527241	LAG1-Interacting Protein

GN, Gene name; AN, Accession Number; γ1 RPKM, the RPKM value of gene in resting Vγ1^+^ γδ T cells; γ4 RPKM, the RPKM value of gene in resting Vγ4^+^ γδ T cells; “*”, Gene’s alternatively spliced transcript variants.

In the PMA/Ionomycin-activated Vγ1^+^ and Vγ4^+^ γδ T cells, we found 20 differentially expressed genes, most of which are related to cytokines, cell differentiation, transcription and translation (Table 4). Activated Vγ1^+^ γδ T cells expressed higher levels of IL-4 and IL-5. Vγ4^+^ γδ T cells secreted more IL-17A and IL-17F. Alternatively spliced transcript variants *Smurf1*, *Pphln1*, *Ilf3* and *Sema6d* were preferentially expressed in Vγ4^+^ γδ T cells. Vγ1^+^ γδ T cells preferentially expressed *Bcl11b*, *Hmga1* and a second spliced transcript variant of *Sema6d*. These results taken together indicate that a very small number of genes are sufficient to define the characteristics of these two subsets of γδ T cells.

**Table 4:** 20 transcripts expressed differently between activated Vγ1^+^ and Vγ4^+^ γδ T cells.

Category	GN	AN	γ1 RPKM	γ4 RPKM	Gene function
Cytokine					
	IL-17A	NM_010552	0.360943	5.13468	Inflammation
	IL-17F	NM_145856	0.0597255	2.86449	Inflammation
	IL −4	NM_021283	3.25878	0.126145	B-cell activation
	IL- 5	NM_010558	3.89427	0.355691	Differentiation of late-developing B-cells
Cell differentiation					
	*BCL11B	NM_021399	0.980076	0.0949643	Regulator of thymocyte development
	SCART2	NM_175533	0.064865	0.562434	Scavenger receptor
	*SMURF1	NM_029438	0.00711406	6.66404	E3 ubiquitin-protein ligase
	*PPHLN1	NM_175363	0.0117361	8.20294	Epidermal integrity and barrier formation
	*SEMA6D	NM_199238	0.18461	6.73579	Neuronal connections
	*SEMA6D	NM_199240	3.96619	0.503894	Neuronal connections
Transcription/Translation					
	*HMGA1	NM_001039356	5.04288	0.0149135	Regulation of inducible gene transcription
	*ILF3	NM_001042707	0.0338755	6.4809	Regulate gene expression
	*ZFP692	NM_001040686	0.0424843	5.96934	Transcriptional regulation
	*GM5633	XM_001480560	27.59	0.0958007	mRNA turnover and ribosome assembly
	*TXNL4A	NM_001042408	0.577893	19.5602	Pre-mRNA splicing
Miscellaneous					
	*DCUN1D2	NM_001042651	0.459734	3.03555	DCN1-Like Protein 2
	*GNAS	NR_003258	23.7675	2.81557	G protein α subunit
	*CEACAM1	NM_001039186	0.870669	0.0859013	Immunoglobulin per family
	*NOLC1	NM_001039353	2.11695	18.0875	Lipid transporter activity
	*PLEC	NM_201392	0.00166738	1.77384	Intermediate Filament Binding Protein
	*SYTL3	NM_183369	0.146591	4.75764	Vesicle trafficking

GN, Gene name; AN, Accession Number; γ1 RPKM, the RPKM value of gene in activated Vγ1^+^ γδ T cells; γ4 RPKM, the RPKM value of gene in activated Vγ4^+^ γδ T cells; “*”, Gene’s alternatively spliced transcript variants.

### Validation of differentially expressed genes in Vγ1^+^ and Vγ4^+^ γδ T cells

**Figure 3:**
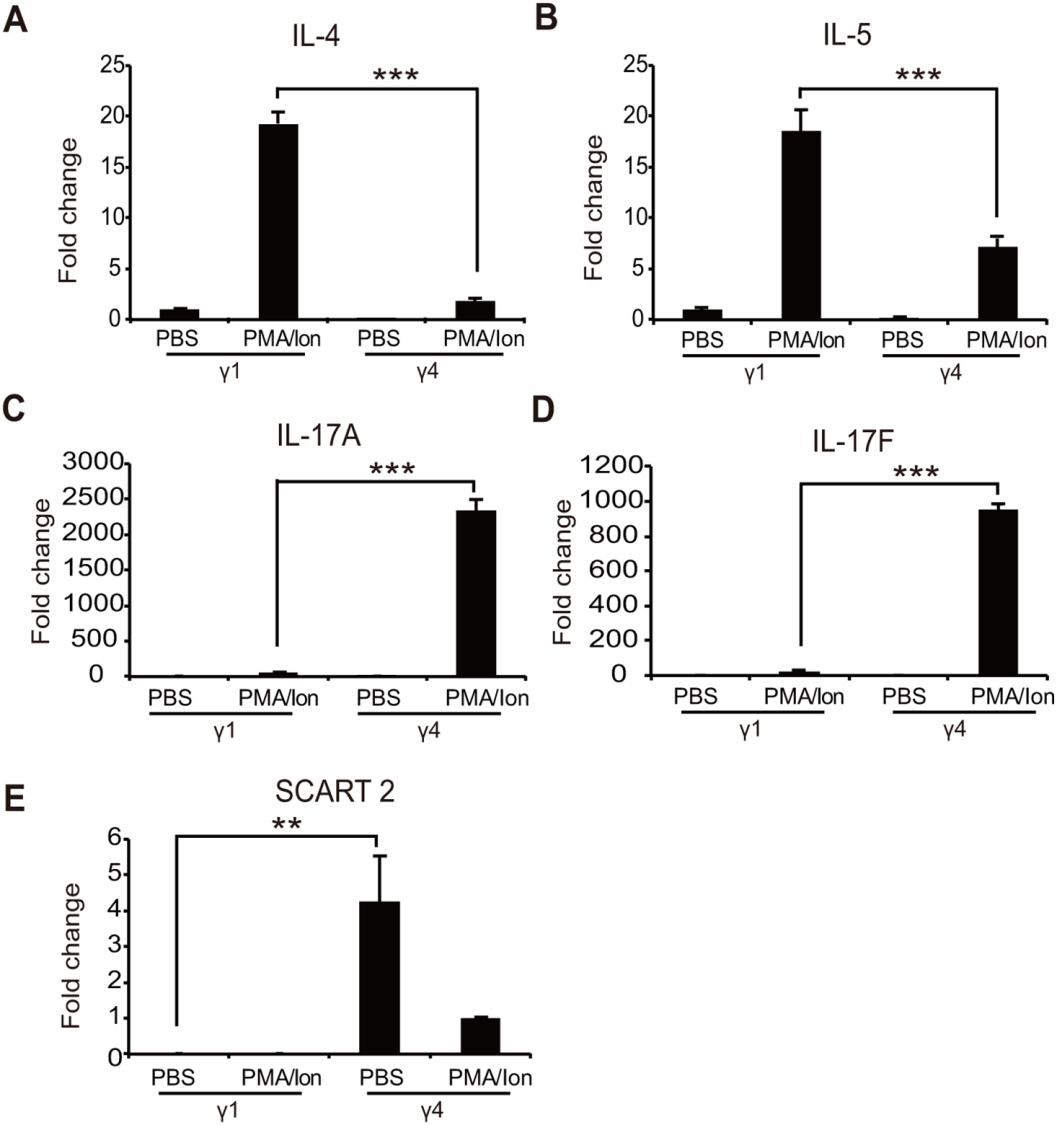
Gene verification with real-time quantitative PCR. Several genes from Vγ1^+^ and Vγ4^+^ γδ T cells were selected for verification against biological replicates using real-time quantitative PCR (A-E). Expression data for each gene were normalized against β-actin. Data shown are the means ± SD (error bars). (* p≤0.05, ** p≤0.01, *** p≤0.001, unpaired two-tailed Student’s t-test). Data are representative of three independent experiments.

**Figure 4:**
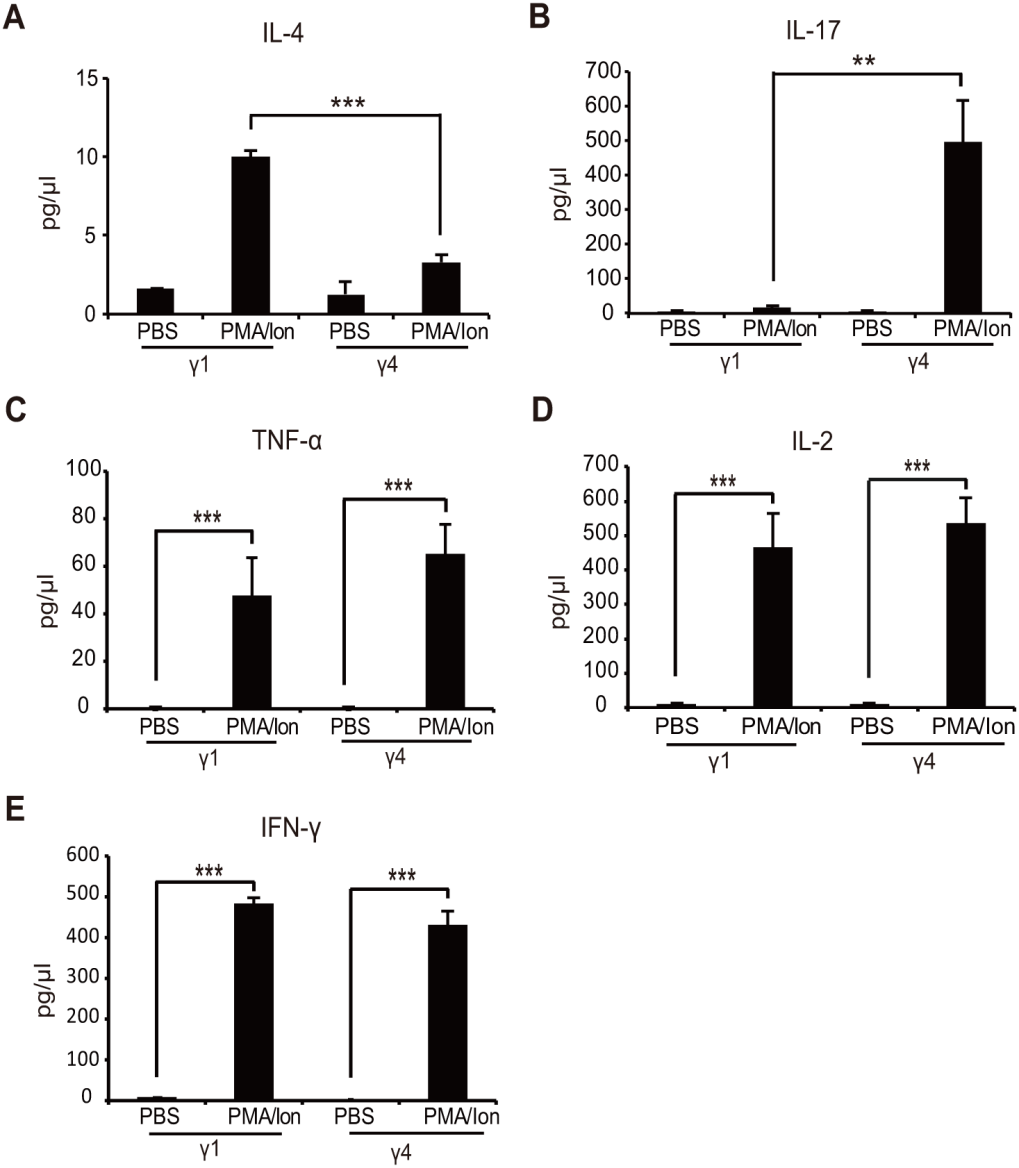
Cytokine expression. ELISA results of (A) IL-4 and (B) IL-17 after PBS or PMA and Ionomycin treatment. MILLIPLEX results of (C) TNF-α, (D) IL-2 and (E) IFN-γ after PBS or PMA and Ionomycin treatment. Data shown are mean ± SD (error bars). (* p≤0.05, ** p≤0.01, *** p≤0.001, unpaired two-tailed Student’s t-test). Data are representative of three independent experiments.

We measured expression levels in both subsets by PCR to verify whether the genes identified via RNA-sequencing were differentially expressed in Vγ1^+^ and Vγ4^+^ γδ T cells. Several genes from both subsets were randomly selected for verification (Figure 3). Consistent with the RNA-seq results, *Scart2* mRNA was only detectable in Vγ4^+^ γδ T cells (Figure 3E). Real-time quantitative PCR confirmed that expression levels of IL-4 and IL-5 mRNA were significantly higher in PMA/Ionomycin-activated Vγ1^+^ γδ T cells compared with activated Vγ4^+^ γδ T cells (Figure 3A and 3B), whereas the expression levels of IL-17A and IL-17F mRNA were significantly higher in activated Vγ4^+^ γδ T cells (Figure 3C and 3D). ELISA results confirmed that IL-4 was mainly expressed in activated Vγ1^+^ γδ T cells whereas IL-17 was predominately expressed in activated Vγ4^+^ γδ T cells (Figure 4A and 4B). Together, all of the genes randomly selected for expression analysis were consistent with RNA-seq results, confirming differential expression in Vγ1^+^ and Vγ4^+^ γδ T cells.

### Gene expression in the resting compared with PMA/Ionomycin-activated state

**Figure 5:**
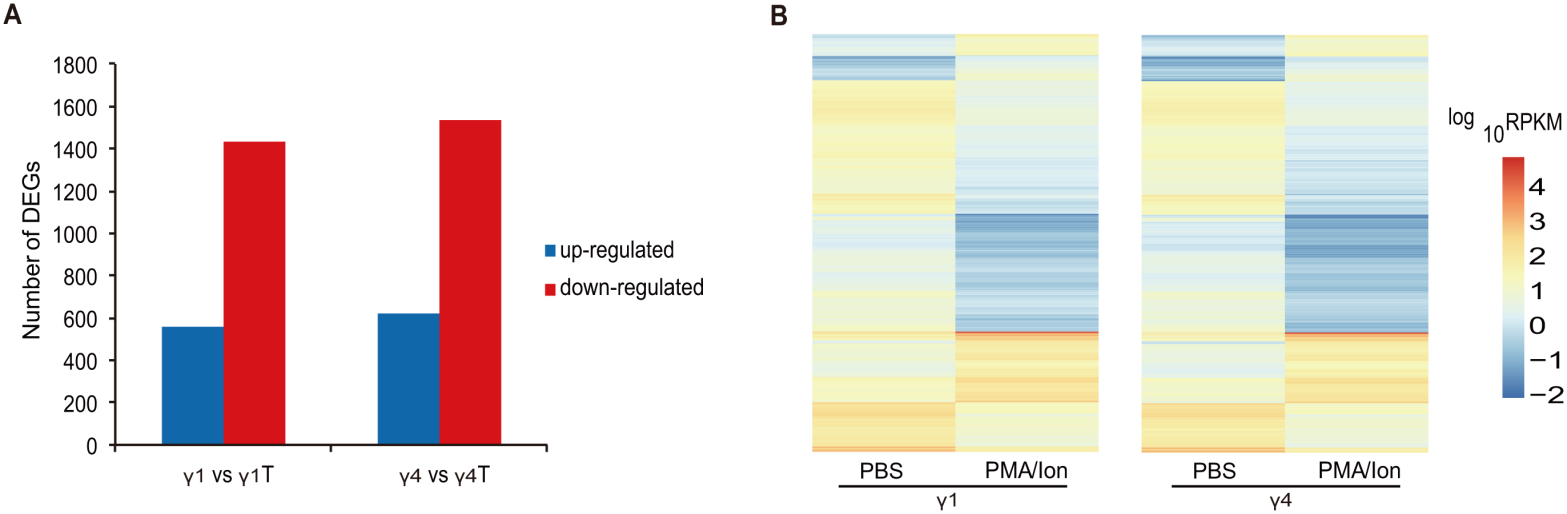
Changes in gene expression profile among Vγ1^+^ and Vγ4^+^ γδ T cells. (A) The number of up and down regulated genes between resting and activated Vγ1^+^ and Vγ4^+^ γδ T cells. (B) Heat maps of 1,995 (Vγ1^+^) and 2,158 (Vγ4^+^) differentially expressed transcripts associated with activated cells using hierarchical clustering analysis. γ1 vs γ1 T, resting Vγ1^+^ vs activated Vγ1^+^ γδ T cells; γ4 vs γ4T, resting Vγ4^+^ vs activated Vγ4^+^ γδ T cells; RPKM, Reads Per Kilo bases per Million reads.

PMA/Ionomycin treatment induces a robust non-TCR mediated response in γδ T cells [18]. As expected, we found both Vγ1^+^ and Vγ4^+^ γδ T cells responded robustly to PMA/Ionomycin treatment, as reflected in the total number of genes that significantly changed in each subset. 1,995 transcripts were differentially expressed between the resting and activated Vγ1^+^ γδ T cells, with 560 up-regulated and 1435 down-regulated genes (q<0.05) (Figure 5A, Dataset S2). 2,158 transcripts were differentially expressed between resting and activated Vγ4^+^ γδ T cells, with 622 up-regulated and 1536 down-regulated genes (q<0.05) (Figure 5A,Dataset S3). For a global perspective on gene dynamics, two heat maps of the 1,995 and 2,158 differentially expressed gene transcripts were generated using hierarchical clustering analysis (Figure 5B).

DAVID functional annotation clustering analysis showed the 1,995 transcripts identified via activation of Vγ1^+^ γδ T cells were enriched for 32 KEGG pathways (p<0.05) (Table 5). 2,158 transcripts identified via activation of Vγ4^+^ γδ T cells were enriched for 29 KEGG pathways (p<0.05) (Table 6). Our comparison of the KEGG pathways between the two subsets showed they share most of the same signal pathways including cytokine-cytokine receptor interaction, Jak-STAT signaling pathway, hematopoietic cell lineage, apoptosis, and pathways in cancer. Interestingly, both Vγ1^+^ and Vγ4^+^ γδ T cells showed connections to the intestinal immune network for IgA production, biosynthesis of unsaturated fatty acids, glycosphingolipid biosynthesis, glutathione metabolism, and purine and pyrimidine metabolism.

**Table 5:** Significantly changed genes between resting and activated Vγ1^+^ γδ T cells enriched for KEGG pathways.

Term	Count	P-Value
Cytokine-cytokine receptor interaction	45	1.50E-05
Jak-STAT signaling pathway	29	3.80E-04
Hematopoietic cell lineage	19	6.80E-04
Glutathione metabolism	14	8.20E-04
Apoptosis	19	1.10E-03
Intestinal immune network for IgA production	14	1.20E-03
Prostate cancer	19	1.60E-03
Small cell lung cancer	18	2.10E-03
Pathways in cancer	46	4.40E-03
p53 signaling pathway	15	4.40E-03
Bladder cancer	11	4.70E-03
Glycosphingolipid biosynthesis	8	5.40E-03
Pyrimidine metabolism	18	7.80E-03
Endometrial cancer	12	8.10E-03
Arrhythmogenic right ventricular cardiomyopathy	15	9.50E-03
Natural killer cell mediated cytotoxicity	21	9.80E-03
One carbon pool by folate	6	1.30E-02
Type I diabetes mellitus	13	1.30E-02
Colorectal cancer	16	1.40E-02
Melanoma	14	1.40E-02
Glioma	13	1.50E-02
Allograft rejection	12	1.80E-02
Chemokine signaling pathway	27	2.00E-02
Phosphatidylinositol signaling system	14	2.20E-02
ABC transporters	10	2.30E-02
Non-small cell lung cancer	11	2.80E-02
Asthma	8	3.10E-02
Insulin signaling pathway	21	3.40E-02
Biosynthesis of unsaturated fatty acids	7	3.70E-02
Fc gamma R-mediated phagocytosis	16	4.10E-02
Graft-versus-host disease	11	4.30E-02
ECM-receptor interaction	14	4.60E-02

Database for Annotation, Visualization and Integrated Discovery (DAVID), was used to analyze biological pathways associated with the differentially expressed gene transcripts. 1,995 transcripts that were identified to be related to the activation of Vγ1^+^ γδ T cells were enriched for 32 KEGG pathways (p<0.05). KEGG, Kyoto Encyclopedia of Genes and Genomes.

**Table 6:** Significantly changed genes between resting and activated Vγ4^+^ γδ T cells enriched for KEGG pathways.

Term	Count	P-Value
Cytokine-cytokine receptor interaction	50	1.00E-06
Biosynthesis of unsaturated fatty acids	10	8.50E-04
Hematopoietic cell lineage	19	1.30E-03
Intestinal immune network for IgA production	14	2.00E-03
Jak-STAT signaling pathway	28	2.00E-03
Pathways in cancer	49	2.90E-03
Prostate cancer	19	3.00E-03
Small cell lung cancer	18	3.90E-03
Colorectal cancer	18	4.40E-03
Arrhythmogenic right ventricular cardiomyopathy	16	6.50E-03
Glycosphingolipid biosynthesis	8	7.30E-03
p53 signaling pathway	15	7.40E-03
Dilated cardiomyopathy	18	8.80E-03
Apoptosis	17	1.10E-02
Endometrial cancer	12	1.20E-02
Chemokine signaling pathway	29	1.30E-02
Non-small cell lung cancer	12	1.60E-02
One carbon pool by folate	6	1.70E-02
Melanoma	14	2.20E-02
Glioma	13	2.30E-02
Amyotrophic lateral sclerosis (ALS)	12	2.40E-02
Pyrimidine metabolism	17	2.80E-02
Glutathione metabolism	11	3.10E-02
Toll-like receptor signaling pathway	17	3.60E-02
Chronic myeloid leukemia	14	3.70E-02
Purine metabolism	24	3.90E-02
Regulation of actin cytoskeleton	31	4.10E-02
Endocytosis	29	4.50E-02
Type I diabetes mellitus	12	4.60E-02

Database for Annotation, Visualization and Integrated Discovery (DAVID), was used to analyze biological pathways associated with the differentially expressed gene transcripts. 2,158 transcripts that were identified to be related to the activation of Vγ4^+^ γδ T cells were enriched for 29 KEGG pathways (p<0.05). KEGG, Kyoto Encyclopedia of Genes and Genomes.

We analyzed the expression levels of some common representative markers in resting Vγ1^+^ and Vγ4^+^ γδ T cells (Table 7). Both subsets expressed high levels of the β and γ chains in the cytokine receptor genes IL-2R, IL-7R and interferon gamma receptor 1. We measured medium expression levels of interferon (alpha and beta) receptor 1 and 2, α and β chains of IL-10R, IL-18 receptor 1, IL-18 receptor beta, IL-21R, α chain of IL-27R, beta receptor II of transforming growth factor and IL-4R. Both Vγ1^+^ and Vγ4^+^ γδ T cells expressed high levels of TGF-β, known to down-regulate immune response and a key regulator of T cell and Th17 differentiation [19]–[21]. Additionally, both Vγ1^+^ and Vγ4^+^ γδ T cells expressed IL-16. In contrast, IFN-γ, TNFα and LTA were expressed at relatively low levels during the resting condition. Several conventional T cell surface antigens were highly expressed in Vγ1^+^ and Vγ4^+^ γδ T cells, including CD2, CD3, CD7, CD27, CD37, CD47, CD48, CD52, CD53, CD82 and CD97. However, some surface markers, including CD25, CD44, and CD69 were expressed at low levels.

**Table 7:** Expression levels for specific genes identified by RNA-seq in both resting Vγ1^+^ and Vγ4^+^ γδ T cells.

	Expression levels			
Category	++++	+++	++	+
Cytokine/chemokine/similar				
	CCL4	XCL1	CCL1	CCR1
	CCL5	Il16	CCL3	CCR10
	CCR2	Ifnar1	CCR7	CCR4
	CCR5	Ifnar2	CXCR4	CCR8
	CXCR3	Il10ra	Csf1	CCRk
	CXCR6	Il10rb	Ifng	CCRl2
	Il2rb	Il18RAP	Tnf	Il18
	Il7R	Il21r	Lta	Tgfb
	Ifngr1	Il27ra	Il12rb1	Il11ra1
	Il2rg	Il4ra	Il15ra	Il15ra
	Tgfb1	TnfrSF1B	Il3ra	Il17rd
		Tgfbr2	Ifnar1	Il1rap
		Il18r1		Il20rb
				Il4i1
Surface antigens				
	Cd2	Cd164	Cd1d1	Cd1d2
	Cd27	Cd247	Cd226	Cd200
	Cd37	Cd96	Cd244	Cd320
	Cd3d	CTLA4	Cd274	Cd38
	Cd3e		Cd28	Cd3eap
	Cd3g		Cd5	Cd55
	Cd47		Cd6	Cd63
	Cd48		Cd68	Cd69
	Cd52		Cd72	Cd74
	Cd53		Cd79b	Cd79a
	Cd7		Cd80	Cd81
	Cd82		Cd84	Cd93
	Cd97		Cd8a	
			Cd8b1	
			Cd9	
			Cd25	
			Cd44	
			Cd62L	
NK cell related				
	Klrc1; NKG2A	KLRK1; NKG2D	Klrb1c; NKRP1A	
	KLRD1; CD94		Klrc2; NKG2C	
	Cd160; BY55		Klrc3; NKG2E	
Integrin				
	ITGB7; Ly69	ITGA4; Cd49D	ITGAX; Cd11c	ITGAD; Cd11d
	ITGB2; Cd18	ITGB3; Cd61	ITGAM; Cd11b	ITGA6; Cd49f
	ITGAL; Cd11a		ITGAV; Cd51	ITGA3; Cd49C
	ITGAE; Cd103			ITGA2; Cd49b
	ITGB1; Cd29			ITGB5
Miscellaneous				
	Gzma	Gzmc	Fasl	Tlr1
	Gzmb	Gzmk		Tlr12
				Tlr6

According to the expression abundance, transcripts with RPKM value over 1 were divided into 4 categories: “+” (1–10 RPKM), “++”(10–50 RPKM), “+++” (50–100 RPKM), and “++++”(>100 RPKM). RPKM, Reads Per Kilo bases per Million reads.

Resting Vγ1^+^ and Vγ4^+^ γδ T cells expressed high levels of Fas ligand and the granzymes *Gzma* and *Gzmb*. NK cells associated receptors including NKG2A, CD94 and NKG2D were also highly expressed by both resting subsets (Table 7). Interestingly, several integrins were highly expressed including *Itgb7* (Ly69), *Itgb2* (Cd18), *Itgal* (Cd11a), *Itgae* (Cd103) and *Itgb1* (Cd29) (Table 7). However, none of the TLRs showed high expression levels in either subset. In fact, TLR1, TLR6 and TLR12 were the only three detected, and with very low expression levels.

PMA/Ionomycin treatment activates Vγ1^+^ and Vγ4^+^ γδ T cells, upregulating T cell activation markers CD25, CD69 and CD44 along with several cytokines. Therefore, we analyzed the expression of these representative markers in activated Vγ1^+^ and Vγ4^+^ γδ T cells. As expected, PMA/Ionomycin treatment induced expression of XCL1, CCL3, CCL4, CCL1, IFN-γ, Lta, Csf2, TNF-α, IL-2, *Gzmb* and *Gzmc* (Table 8). MILLIPLEX results further confirmed that both Vγ1^+^ and Vγ4^+^ γδ T cells produced high levels of TNF-α, IL-2 and IFN-γ after PMA/Ionomycin treatment (Figure 4C, 4D and 4E). This is consistent with the hypothesis that γδ T cells acquire a pre-activated status poised to actively transcribe genes related to effector functions. Interestingly, IL-10, a Th1 cytokine down-regulator, was also highly expressed by both Vγ1^+^ and Vγ4^+^ γδ T cells (Table 8).

**Table 8:** Expression levels of significantly changed genes identified by RNA-seq in both Vγ1^+^ and Vγ4^+^ γδ T cells after PMA/Inomycin treatment.

	Expression levels				
Category	++++	+++	++	+	−
Cytokine/chemokine/similar					
	XCL1	CCL9	CXCR3	CXCL14	CXCR4
	CCL3	Tnfsf11	CCR2	CCR7	CCR8
	CCL4	Il2	Il13	Ifngr1	CCR10
	CCL1	Tnfrsf8	Tnfrsf12a	Tnfaip8l2	CX3CR1
	Ifng	Tnfsf9	Ifnar1	Il10rb	Tnfrsf11b
	Tnfrsf9		Vegfa	Il1rl1	Tnfrsf23
	Lta			Il1r2	Tnfrsf26
	Csf2			Ifnar2	Tgfb1i1
	Tnfa			Il16	Il11ra1
	Tnfsf14			Il10ra	Tnfrsf13c
	Tnfrsf4			Il1rap	Tnfsf12
	Il10			Il7r	Il17rd
				Il1rl1	il-18
				Il33	
				Tnfaip8l1	
Surface antigens					
	Cd44	Cd274	Cd63	Cd83	Cd1d1
	Cd25	Cd7	Cd96	Cd24a	Cd200r1
		Cd69	Cd320	Cd79b	Cd79a
			Cd70	Cd93	Cd1d2
					Cd200r4
					Cd55
NK cell related					
			KLRD1	Klrb1c	Klrb1d
Miscellaneous					
	Gzmb		Gzme	Gzmk	Tlr1
	Gzmc		Gzmf		Tlr6
					Tlr12

According to the expression abundance, transcripts were divided into 5 categories: “−” (<1 RPKM), “+” (1–10 RPKM), “++”(10–50 RPKM), “+++” (50–100 RPKM), and “++++”(>100 RPKM). RPKM, Reads Per Kilo bases per Million reads.

We analyzed the expression levels of transcription factors related to Th cell differentiation and cytokine secretion (Dataset S4). Both Vγ1^+^ and Vγ4^+^ γδ T cells expressed high levels of *Gata3*, *T-bet*, *Eomes*, *Foxp1*, *Stat1*, *Stat3*, *Stat4*, *Stat5a*, *Stat5b*, *Stat6*, *Runx3*, *Irf1*, *Ikzf1*, *Ikzf3*, *Ets1*, *Junb* and *Batf* at resting condition. After PMA/Ionomycin treatment, the expression levels of *Stat5a* and *Irf4* were upregulated significantly. The expression levels of *T-bet*, *Eomes*, *Foxp1*, *Stat5b*, *Gfi1* and *Junb* were upregulated slightly. Interestingly, the expression levels of *Gata3*, *Irf4* and *Gfi1* were slightly higher in Vγ1^+^ γδ T cells than Vγ4^+^ γδ T cells after PMA/Ionomycin treatment.

**Figure 6:**
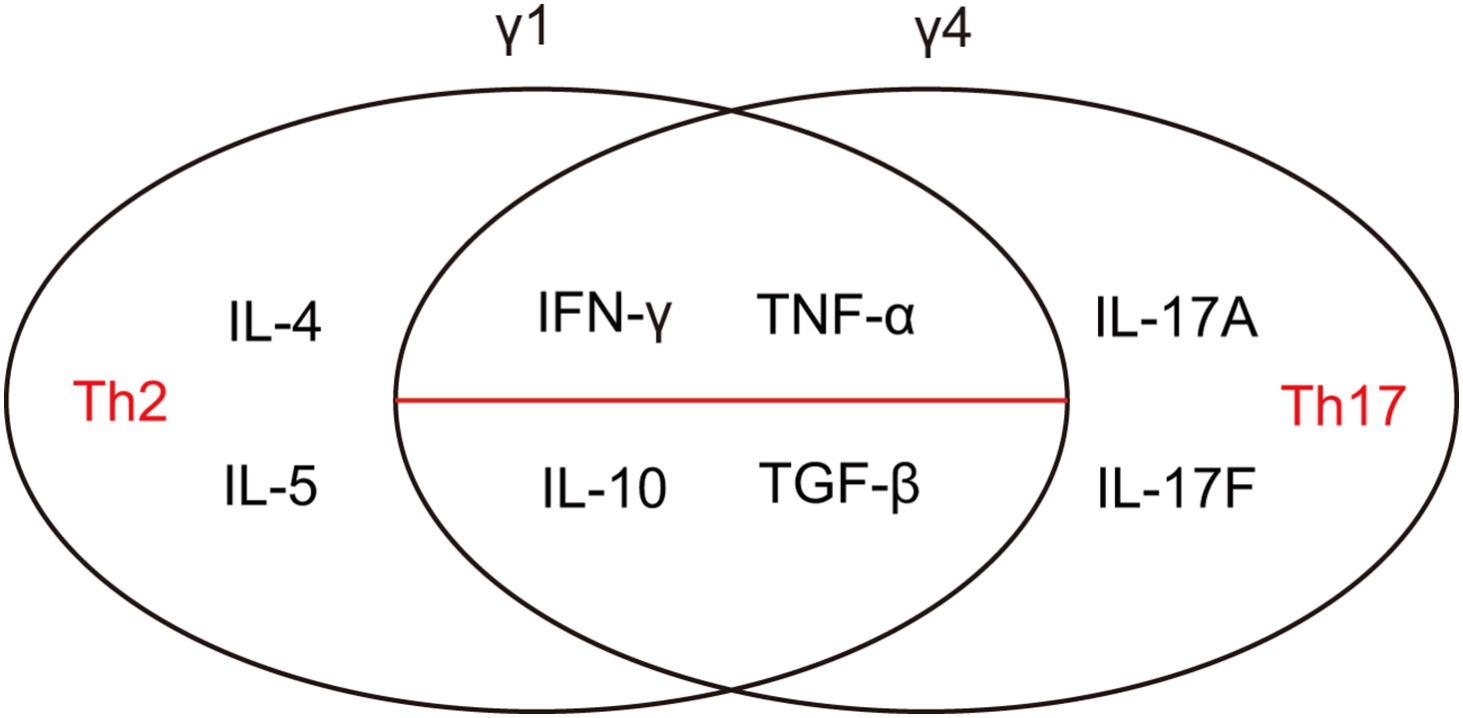
Cytokines secreted by Vγ1^+^ and Vγ4^+^ γδ T cells. Both subsets of γδ T cells produce IFN-γ, TNFα, TGF-β and IL-10. Vγ1+ γδ T cells tend to produce Th2 type cytokines IL-4 and IL-5 while Vγ4^+^ γδ T cells tend to produce IL-17.

Taken together, these findings indicate that both Vγ1^+^ and Vγ4^+^ γδ T cells maintain phenotypes producing IFN-γ, TNFα, TGF-β and IL-10. However, Vγ1^+^ γδ T cells tend to produce Th2 type cytokine while Vγ4^+^ γδ T cells preferentially produce IL-17 (Figure 6).

## Discussion

Phylogenetic analysis suggests γδ T cells are precursors to modern B and αβ T cells [22]. γδ T cells are divided into subsets based on composition of T cell receptors. Interestingly, γδ T cell subsets demonstrate bias in carrying out particular functions [1]. Previously, Jutila et al. analyzed gene expression profiles of bovine CD8^+^ and CD8^−^ γδ T cells using microarray and serial analysis of gene expression (SAGE) technology. They concluded inherent gene expression differences in subsets defined their distinct functional responses [23], [24]. In addition, Kress et al. found considerable inherent differences in gene expression among subsets of post PMA/Ionomycin or LPS treatment of circulating Vδ1 and Vδ2 subsets in humans [18].

Vγ1^+^ and Vγ4^+^ γδ T cells are major subpopulations of peripheral γδ T cells in mice. Although global gene expression profiles of all emergent γδ thymocyte subsets have been reported by the Immunological Genome (ImmGen) Project and much knowledge has been obtained about the early divergence of gene expression programs between different γδ thymocyte subsets [12], a comprehensive gene expression profiles analysis of peripheral Vγ1^+^ and Vγ4^+^ γδ T cells isn’t available. A major hurdle has been the limited number of cells that can be obtained from healthy mice.

In this study, we resolved the limited cell count issue by establishing a primary culture method expanding Vγ1^+^ and Vγ4^+^ γδ T cells simultaneously from a single pool of mouse splenocytes. Our results proved that *in vitro* TCR-induced expansion for a week did not significantly change the proportion of Vγ1^+^ and Vγ4^+^ γδ T cells. We provide a comprehensive gene expression profile of mouse peripheral Vγ1^+^ and Vγ4^+^ γδ T cells in the resting and activated state. Although Vγ1^+^ and Vγ4^+^ γδ T cells share similar transcript profiles, we identified subset specific genes defining characteristics of each subset.

We identified 24 transcripts differentially expressed in resting Vγ1^+^ and Vγ4^+^ γδ T cells, and 20 transcripts differentially expressed after PMA/Ionomycin treatment. Consistent with γδ thymocytes, expression levels of *Rorc*, *Sox13* and *Scart2* were higher in Vγ4^+^ γδ T cells compared with Vγ1^+^ γδ T cells [12]. *Rorc* expression is reported in γδ T cells, Th22 cells, NKT cells, CD4^+^ CD8^+^ thymocytes, and others that do not belong to the T or B cell lineage [25]–[28]. *Rorc* is recognized as a lineage-specific transcription factor of Th17 and is also required for IL-17 production [29]. Transcription factor *Sox13* serves a general role in the differentiation of γδ T cells [30]. Moreover, Gray et al. reported that *Sox13* was indispensable for the maturation of Vγ4^+^ Th17 cells [31], [32]. Scavenger receptor *Scart2* is a marker of γδ T cells prepared to secrete IL-17A [12], [31], [33], [34]. Our data showing Vγ4^+^ γδ T cells compared with Vγ1^+^ γδ T cells produce significantly more IL-17A and IL-17F after PMA/Ionomycin treatment are also consistent with findings in γδ thymocytes [12]. Our findings show Vγ1^+^ γδ T cells produce significantly more IL-4 and IL-5 after PMA/Ionomycin treatment compared with Vγ4^+^ γδ T cells. This finding is consistent with earlier reports showing Vγ1^+^ γδ T cells preferentially produce IL-4, and the depletion of Vγ1^+^ subset cells increases host resistance against *Listeria monocytogenes* infection [35]. It is important to note that Vγ1^+^ γδ T cells suppress Vγ4^+^ γδ T cell mediated antitumor function through IL-4 [36].

Alternative splicing plays an important role in increasing functional diversity of eukaryotes. Compared with the ImmGen Project, one of the advantages of RNA-seq is able to quantify individual transcript isoforms and identify differentially expressed transcripts between Vγ1^+^ and Vγ4^+^ γδ T cells. We found *Bclaf1* and *Atf2* were preferentially expressed in Vγ4^+^ γδ T cells while *Hmga1* and *Bcl11b* were preferentially expressed in Vγ1^+^ γδ T cells. As a transcriptional repressor, *Bclaf1* interacts with several members of the *Bcl2* protein family and plays a role in the regulation of apoptosis and DNA repair [37], [38]. *Bclaf1* also plays an important role in lymphocyte homeostasis and activation [39]. *Atf2* transcription factor is a member of the leucine zipper family of DNA binding proteins and forms a homodimer or a heterodimer with *c-Jun*, stimulating cAMP responsive element (CRE) dependent transcription. *Atf2* expression is lower in CD8^+^ T cells compared with CD4^+^ T cells, a functional explanation to the differential response to glucocorticoids between CD8^+^ and CD4^+^ T cells [40]. As an architectural chromatin factor, *Hmga1* binds preferentially to the minor groove of AT rich regions in double stranded DNA. It is involved in many cellular processes including regulation of inducible gene transcription, insulin resistance, diabetes and malignant transformation [41], [42]. Nakao et al. revealed a new role for *Hmga1* in transcriptional silencing in T cell lineages and leukemic cells [43]. However, the roles of *Bclaf1*, *Atf2* and *Hmga1* in γδ T cells have not been reported. *Bcl11b* is a T-cell specific gene and required for T-lineage commitment. Aberrant expression of *Bcl11b* contributes to human T-ALL [44]. In contrast with ImmGen Project results showing *Bcl11b* was preferentially expressed in Vγ4^+^ γδ thymocytes [12], we identified one transcript isoform of *Bcl11b* preferentially expressed in activated Vγ1^+^ γδ T cells. The role of the transcript isoform of *Bcl11b* in Vγ1^+^ γδ T cells needs further study.

Many of the differentially expressed gene transcripts identified in activated Vγ1^+^ and Vγ4^+^ γδ T cells shared similar signaling pathways. We found higher expression of IL-4 and IL-5 in activated Vγ1^+^ γδ T cells. This suggests a role in asthma given that Vγ4^+^ γδ T cells suppress airway hyperresponsiveness, compared with Vγ1^+^ γδ T cells that enhance airway hyperresponsiveness and raise levels of Th2 cytokines and eosinophils infiltration in the airways [8], [9], [45].

Both resting Vγ1^+^ and Vγ4^+^ γδ T cells exhibited high levels of transcripts for several chemokines and chemokine receptors, including CCL4, CCL5, CCR2, CCR5 and CXCR3. These data highlight the role of Vγ1^+^ and Vγ4^+^ γδ T cells in immunoregulatory and inflammatory processes. For example, CCL4 (MIP-1beta) and CCL5 (RANTES) are both Th1-associated chemokines that bind to CCR5. Up-regulation of CCR5 ligands may play a role in the recruitment process of blood monocytes, memory T helper cells and eosinophils. CCR2 is expressed on both Vγ1^+^ and Vγ4^+^ γδ T cells, and is necessary for the accumulation of γδ TILs to the tumor bed [46]. It is interesting to note that CXCR6 was previously thought to be expressed in human Vδ2 cells, but not Vδ1 cells [18]. However, we found high CXCR6 levels in both Vγ1^+^ and Vγ4^+^ γδ T cells. CXCR6 plays a critical role in NK cell memory of haptens and viruses [47]. Whether CXCR6 plays a role in Vγ1^+^ and Vγ4^+^ γδ T memory cells needs further examination.

Integrins play key roles in immune responses, leukocyte trafficking and many human diseases. Most integrin related research has been focused on αβ T cells, with little published on γδ T cells. Our results show several integrins were highly expressed in Vγ1^+^ and Vγ4^+^ γδ T cells. For example, *Itgae* (Cd103), implicated in epithelial T cell retention, is highly expressed on Vγ1^+^ and Vγ4^+^ γδ T cells [48]. *Itgae* contributes to clustering and activation of Vγ 5 TCRs expressed by epidermal T cells [49]. Signals mediated by integrins play important roles in the activation of T cells [50]. Therefore, we suggest stimulating integrin expression provides a costimulation signal, increasing the sensitivity of γδ T cell activation.

PMA/Ionomycin induces a robust non-TCR mediated response in Vγ1^+^ and Vγ4^+^ γδ T cells. We show after PMA/Ionomycin treatment several activation markers of T cells were upregulated including CD25, CD69 and CD44, along with most cytokine genes in both subsets. In addition, activated Vγ1^+^ and Vγ4^+^ γδ T cells produced high levels of XCL1, CCL3, CCL4, CCL1, IFN-γ, TNFα, Lta, Csf2 and IL-10. IFN-γ and TNFα are Th1 type cytokines. Previous reports show Vγ4^+^ γδ T cells are the major γδ T subset producing IFN-γ, and they steer CD4^+^ T cells toward a dominant Th1 cell response [7], [51], [52]. Moreover, He et al. reported that CD44 rich Vγ4^+^ γδ T cells produced significantly more IFN-γ compared with Vγ1^+^ γδ T cells, partly due to the high expression level of eomesodermin [16]. In contrast, Matsuzaki et al. reported that Vγ1^+^ γδ T cells were the major γδ T subset producing IFN-γ in response to *L. monocytogenes* infection [53]. The opposing results are likely due to different disease models and treatment methods. A separate study reported higher levels of IL-10 in human Vδ1 cells compared with Vδ2 cells [18]. However, our results show both Vγ1^+^ and Vγ4^+^ γδ T cells produce high levels of IL-10.

Narayan et al. reported that Vγ4^+^ γδ thymocytes expressed high levels of *Stat4*, *Maf*, *Gata3* and *Eomes* compared with Vγ1^+^ γδ thymocytes [12]. However, our results show both Vγ1^+^ and Vγ4^+^ γδ T cells expressed high levels of these transcription factors and the levels of *Gata3* were slightly higher in Vγ1^+^ γδ T cells compared with Vγ4^+^ γδ T cells after PMA/Ionomycin treatment. *Gata3* is critical for Th2 cell differentiation and required for IL-4 production. The higher level of *Gata3* expression in Vγ1^+^ γδ T cells is consistent with the phenotype of Vγ1^+^ γδ T cells producing more IL-4 than Vγ4^+^ γδ T cells. T-bet is a major factor for Th1 cell differentiation and IFN-γ production [54]. *Eomes* is also involved in Th1 differentiation and IFN-γ production [55]. The upregulation of *T-bet* and *Eomes* is consistent with the phenotype of both Vγ1^+^ and Vγ4^+^ γδ T cells that produce high levels of IFN-γ. The difference between our results with the ImmGen Project may be due to the source of γδ T cells. The cells used in the ImmGen Project are γδ thymocytes, however the cells in our study were peripheral γδ T cells derived from the spleen.

Taken together, this study shows both Vγ1^+^ and Vγ4^+^ γδ T cells maintain inflammatory and regulatory phenotypes. Both demonstrate an inflammatory cell phenotype via IFN-γ and TNFα expression. And, both display a regulatory cell phenotype via TGF-β and IL-10 production. Vγ1^+^ γδ T cells produced more Th2 type cytokines, while Vγ4^+^ γδ T cells tended to produce more IL-17. Thus, Th2 type cytokines may explain how Vγ1^+^ γδ T cells affect anti-inflammatory functions in different infection models, and describe the enhancing effect on airway hyperresponsiveness (AHR) [56]. IL-17 cytokines support the pro-inflammatory function of Vγ4^+^ γδ T cells in the infection models and the inhibitory effect on airway hyperresponsiveness (AHR). Although this study was performed in Vγ1^+^ and Vγ4^+^ γδ T cells expanded *in vitro*, which may not fully represent the true status of Vγ1^+^ and Vγ4^+^ γδ T cells *in vivo*, our results support the hypothesis that distinct γδ TCR types direct cells to acquire a certain type of functional programming during thymic development [57].

Complementary to the ImmGen Project, this report provides a comprehensive gene expression profile of mouse peripheral Vγ1^+^ and Vγ4^+^ γδ T cells following PMA/Ionomycin treatment. Although both γδ T cell populations have similar transcript profiles, subset-specific transcripts define distinct characteristics and describe the inherent differences between Vγ1^+^ and Vγ4^+^ γδ T cells.

## Supporting Information

Dataset S1 **Raw data and differential expression analysis in RNA-seq.**
(XLSX)

Dataset S2 **Differentially expressed genes between the resting and activated Vγ1^+^ γδ T cells.**
(XLSX)

Dataset S3 **Differentially expressed genes between the resting and activated Vγ4^+^ γδ T cells.**
(XLSX)

Dataset S4 **Transcription factors related to Th cell differentiation and cytokine secretion.**
(XLSX)
